# Design of a high-precision and non-contact dynamic angular displacement measurement with dual-Laser Doppler Vibrometers

**DOI:** 10.1038/s41598-018-27410-4

**Published:** 2018-06-14

**Authors:** Lei Chen, Dengwei Zhang, Yilan Zhou, Cheng Liu, Shuangliang Che

**Affiliations:** Zhejiang University, State Key Laboratory of Modern Optical Instrumentation, Hangzhou, Zhejiang, 310027 China

## Abstract

A wide dynamic range, high precision, non-contact and large bandwidth angular displacement measurement (ADM) is greatly necessary for the applications such as industrial control and military equipment. This paper proposes a simple and effective ADM method based on Doppler Effect, heterodyne detection and diffuse reflection, which can fulfill these requirements simultaneously. Two beams of parallel light generated by a pair of laser Doppler vibrometers are incident upon the surface of rotational target, then data processing unit acquires the velocity of dual laser incident points on the moving target, and resolves the rotational angular displacement and translation displacement of target through the relationship between dual laser beams dynamically. Several major measurement errors that may affect the ADM accuracy are analyzed. A high precision rotary table is used as an angular displacement standard to verify the measurement range and accuracy, the verification experiment shows that the measurement range is not less than ±10° and the measurement accuracy is 0.0362° based on the method. After using a polynomial error compensation, the measurement accuracy can be promoted to 0.0088°, and this compensation method can be applied to real time measurement.

## Introduction

Angular displacement measurement (ADM) plays an important role in fields of industrial application and scientific research. Many kinds of military equipments, such as aviation and aerospace, radar, robotic, need high precision and dynamic ADM widely; in industrial control and manufacturing field, such as the calibration of robot, the accurate measurement of rotational angular displacement in automatic control and the calibration of IMU (inertial measurement unit), ADM also has great requirements. These application fields need similar ADM with wide dynamic range, high precision, non-contact and wide bandwidth. It’s difficult to meet these requirements simultaneously.

Nowadays, many methods for ADM have been proposed to fulfill industrial demand. Circular optical grating is used to measure rotary angular displacement^1,2^. This method becomes very popular due to high angular displacement resolution (0.001″), good stability and high accuracy (0.05″). With these advantages, circular optical grating is used as an ADM standard for angular displacement calibration^3^. However, circular optical grating has to be fixed at the center of rotary table, so it is inconvenient to be used in non-contact situation. To overcome these disadvantages, Talbot Effect is used for ADM^4^. The basic principle is that different angular displacement of incident light to Talbot Grating corresponds to different light intensity. Image sensor based on Talbot Grating is applied to obtain the intensity and angle information of the light reflected from the object to reconstruct the three-dimensional light field of the object^5–7^. This technique can realize non-contact measurement, however, the chip’s manufacturing technic, sensor array density and reconstruction algorithm restrict the angle measurement range and resolution. Besides, it is used for static image acquisition and cannot realize dynamic and wide bandwidth measurement. With the development of laser interferometry detection, many researchers recommend to use laser interferometry for ADM^8–11^. By detecting the phase difference between reference beam and measurement beam caused by rotation of object, laser interferometry can realize accurate ADM. This method^12^ can get ADM resolution to 0.2° and measurement range up to $\pm 8.5^\circ$. In such systems, a pair of rectangular prisms are needed to be fixed on the object to improve the measurement accuracy, which will increase the load of the object, affect the measurement. Another method, which is based on laser displacement sensors, represents a new way of ADM^13^. They measure the displacement difference between two sensors and resolve the angular displacement with the triangular displacement relationship during rotational. They realize ADM range of $-6^\circ  \sim +\,10^\circ$, ADM accuracy of 0.085°, but the bandwidth of the system is not very wide, just about 400 hz. Based on Homodyne interferometry, Doppler Effect and the geometry relationship between the velocity measured by multiple laser beams and the rotational velocity of shaft, the optical geometry and algorithm of laser torsional Doppler vibrometer is proposed^14,15^. The method is direction and surface shape insensitive, and can realize non-contact measurement. Laser Doppler vibrometer (LDV) has its origins in fluid velocity measurements reported by Yeh and Cummins at Columbia University in 1964^16^. Scanning heads are added to single beam instruments to measure the modality of a structure^17^. A pair of laser beams enables the classic differential measurement in which the relative velocity between two parts of a structure or device is determined^18^. Torsional vibration measurement with a parallel beam configuration is an established and successful use of LDV with applications including torsional damper health, electric machines, railway wheelsets, backlash in gears and crankshaft bending and driveshaft vibration^19–21^. However, torsional Doppler vibrometer also have their limitation and disadvantages. For a commercially available laser torsional Doppler vibrometer RLV-5500 provided by Polytec, datasheet shows that its angular displacement relative measurement error is 2% (measurement range of ±10°), which means its absolute measurement error is just 0.2°. But its measurement accuracy of RPM (Revolution(s) Per Minute) is 0.2%, so laser torsional Doppler vibrometer is always applied to measuring the stability of rotational speed of shaft. However, the fixed standoff distance, fixed parallel beam spacing and low measurement accuracy restrict the application of this instrument.

Currently, all the ADM methods have their own advantages and disadvantages, but they cannot meet the requirements of high dynamic range, high precision, non-contact and wide bandwidth simultaneously. In this paper, a novel and simple ADM method based on dual-laser Doppler vibrometers (d-LDVs) is presented. With laser Doppler interferometry and diffuse reflection, non-contact detection can be realized. In Section 2, the related theory is analyzed and the error equation is derived. In Section 3, an experimental demonstration has been established to verify the measurement accuracy and dynamic range. A compensation method to get more accurate measurement result is presented in Section 3, too.

## Principles and Analysis

Laser Doppler vibrometer (LDV) is a technology for accurate and non-contact measurement of velocity in industrial and metrological applications^22^. It is an instrument utilising Doppler Effect and heterodyne detection^15^. In optic Doppler Effect, light frequency is shifted by instantaneous velocity of the object^23^, therefore the intensity of the interference signal is modulated by velocity of the moving target. The interference signal detected by photodetector is given as follow:

1 $${\rm{I}}({\rm{t}})={{\rm{I}}}_{DC}+k{A}_{1}{A}_{2}\,\cos (2\pi ({f}_{0}-{f}_{D})t+{\phi }_{0})$$

where ${{\rm{I}}}_{DC}$ is DC component, $k$ is constant, ${A}_{1}\,$and ${{\rm{A}}}_{2}$ is the amplitude of measurement light wave and reference light wave on the photodetector respectively, ${f}_{0}\,$is the frequency shift of reference light wave modulated by Bragg acousto-optic modulator, and ${\phi }_{0}$ is the initial phase difference between measurement light wave and reference light wave. The key variable is ${f}_{D}$, known as Doppler shift, whose expression is given below:

2 $${f}_{D}=\frac{2v}{\lambda }$$

where ${\rm{\lambda }}$ is laser wavelength, and $v$ represents velocity of moving target^24^.

Based on their non-contact nature, LDV do not destroy the structure to be measured. Laser Vibrometry is a very sensitive optical technique capable of measuring sub-nanometer or even sub-picometer displacement from near DC to Mhz. In addition to their wide frequency range, LDV has dynamic range not matched by other sensors^23^. With these advantages, we consider applying LDV to dynamic and non-contact ADM. In next sections, the principle and geometry structure of the proposed ADM method with d-LDVs will be illustrated, and the factors that may cause measurement errors will be derived, too.

### Principle and geometry structure of the proposed ADM method with d-LDVs

**Figure 1:**
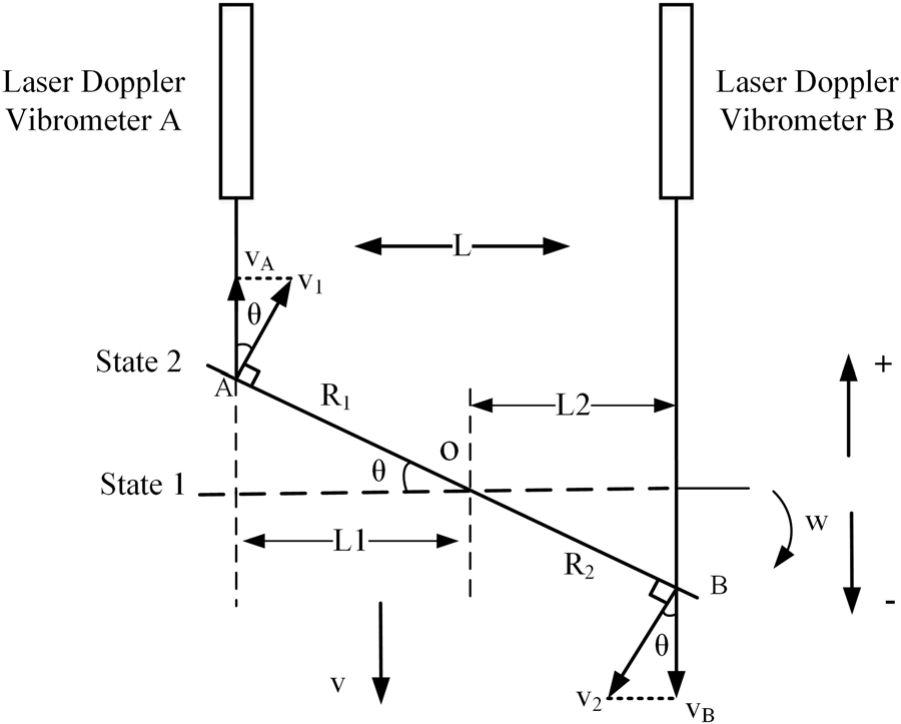
The schematic diagram of dual laser Doppler vibrometers ADM system.

Based on d-LDVs, we designed a dynamic ADM system according to the trigonometric relation when a target is rotational. Figure 1 shows the schematic diagram of the d-LDVs ADM system.

Two beams of parallel light generated by a pair of laser Doppler vibrometers A and B are incident upon the surface of rotational target. When laser beam is incident upon the surface of object, there will be reflection light in all directions due to diffuse reflection, LDV acquires backscattering light along direction of incident laser beam. By diffuse reflection, the proposed system can realize non-contact ADM.

Assuming the object is in a composite motion of rotation and translation. The translation speed of the object is $v$. The object rotates with instantaneous angular velocity ${\rm{\omega }}$ and turns a small angle $\theta$ from state 1 to state 2 around the point o in clockwise, *t* represents the time difference from state 1 to state 2. Suppose ${v}_{A}$ and ${v}_{B}$ represent the instantaneous measurement velocity measured by laser Doppler vibrometers A and B respectively, and L is the distance between dual laser Doppler vibrometers. The direction of speed is specified as follows: when the object is moving away from LDV, the velocity is negative. Then, tangential velocity of laser incident point A and B can be given by

3 $$\{\begin{array}{c}{v}_{1}=\omega \cdot {R}_{1}\\ {v}_{2}=\omega \cdot {R}_{2}\end{array}$$

According to the geometrical relationship shown in Fig. 1, instantaneous measurement velocity measured by laser Doppler vibrometers A and B is a component of the tangential velocity ${v}_{1}$ and ${v}_{2}$ in the direction of the laser beam, which can be given by

4 $$\{\begin{array}{l}{v}_{A}={v}_{1}\cdot \,{\cos }\,\theta +v=\omega \cdot {R}_{1}\cdot \,{\cos }\,\theta +v\\ {v}_{B}=-\,{v}_{2}\cdot \,{\cos }\,\theta +v=-\,\omega \cdot {R}_{2}\cdot \,{\cos }\,\theta +v\end{array}$$

In the meantime, we have the relationship shown as followed:

5 $$\{\begin{array}{c}{L}_{1}={R}_{1}\cdot cos\theta \\ {L}_{2}={R}_{2}\cdot cos\theta \end{array}$$

6 $$L={L}_{1}+{L}_{2}$$

Substituting equation (5) into equation (4), we have

7 $$\{\begin{array}{l}{v}_{A}=\omega \cdot {L}_{1}+v\\ {v}_{B}=-\,\omega \cdot {L}_{2}+v\end{array}$$

Then, substituting equation (7) into equation (6), we have

8 $$\omega =\frac{{v}_{A}-{v}_{B}}{L}$$

Integral instantaneous angular velocity ${\rm{\omega }}$ can obtain angular displacement $\theta$ of object

9 $$\theta ={\int }_{0}^{{t}_{0}}\omega dt={\int }_{0}^{{t}_{0}}\frac{{v}_{A}-{v}_{B}}{L}{\int }_{0}^{{t}_{0}}dt=\frac{{\int }_{0}^{{t}_{0}}{v}_{A}dt-{\int }_{0}^{{t}_{0}}{v}_{B}dt}{L}$$

And the translational displacement *s* can be given by

10 $$s={\int }_{0}^{{t}_{0}}vdt={\int }_{0}^{{t}_{0}}{v}_{A}dt-\theta \cdot {L}_{1}$$

This measurement method is insensitive to surface shape and translation, and has the advantages of non-contact and large bandwidth (up to 10 khz). It is a flexible measurement system, researchers can adjust the parallel beam spacing and measurement distance according to the different measurement needs. The system can obtain angular displacement and translational displacement simultaneously from a composite motion of rotation and translation. The system requires two laser beams to be parallel to each other and the rotating axis of object be perpendicular to the plane where parallel beams are.

### Light intensity non-uniformity error

LDV performs velocity measurement by receiving diffuse light from the surface of the object, as well as ADM system with d-LDVs. The characteristic of diffuse reflection and rotational angular displacement of the object will affect the final detection results. Considering an object as an ideal diffuse reflection source, known as Lambertian light source, the diffuse reflection intensity follows the cosine law of the radiation source with the change of angular displacement $\theta$ between the observation direction and the surface normal.

**Figure 2:**
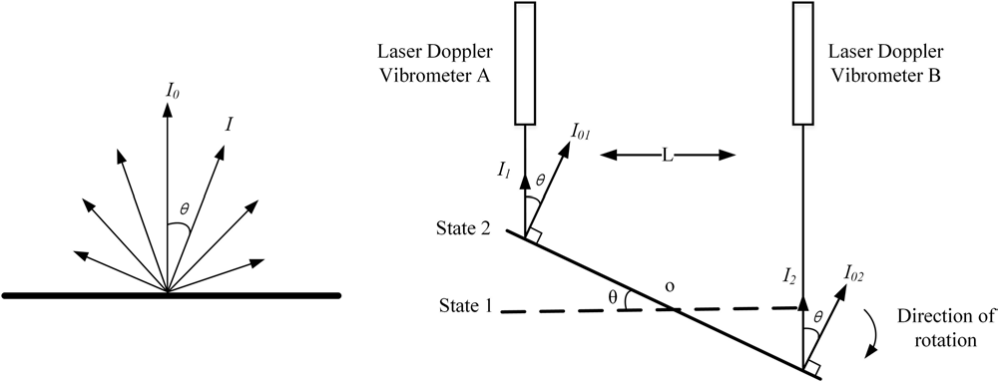
Light intensity non-uniformity error.

The left figure in Fig. 2 depicts Lambertian light source, and the light intensity in any direction can be given by:

11 $$I={I}_{0}\cdot \,\cos \,\theta $$

Lambertian radiation characteristics are suitable for ADM system with d-LDVs. When the object turns an angular displacement $\theta$, light intensity received by dual LDVs decreases with $\cos \,\theta$ at the mean time, as shown in right figure of Fig. 2. Hence, the interference signal detected by photodetector is modulated:

12 $$I({\rm{t}})={I}_{DC}+k{A}_{1}\cdot \sqrt{{\cos }\,\theta }{A}_{2}\cdot \,\cos (2\pi ({f}_{0}-{f}_{D})t+{\phi }_{0})$$

The light intensity received by the detector is weakened, as the noise intensity does not change, the signal to noise ratio (SNR) will be weakened. Modulated SNR can be given by:

13 $${\rm{SNR}}=\frac{\eta {I}_{s}}{hv{\rm{\Delta }}f}cos\theta $$

where $\eta$ is the quantum efficiency of the photodetector, ${I}_{s}$ is the intensity of measurement light signal, $h$ is Planck constant, $v$ is the frequency of the laser, ${\rm{\Delta }}f$ is the bandwidth of system, and $\theta$ is the rotational angular displacement of the object. The rotation of the object and uneven reflectivity of the target surface will lead to the non-uniformity of the received light, which results in the decrease of signal-to-noise ratio.

### Dual parallel laser beams misalignment Error

**Figure 3:**
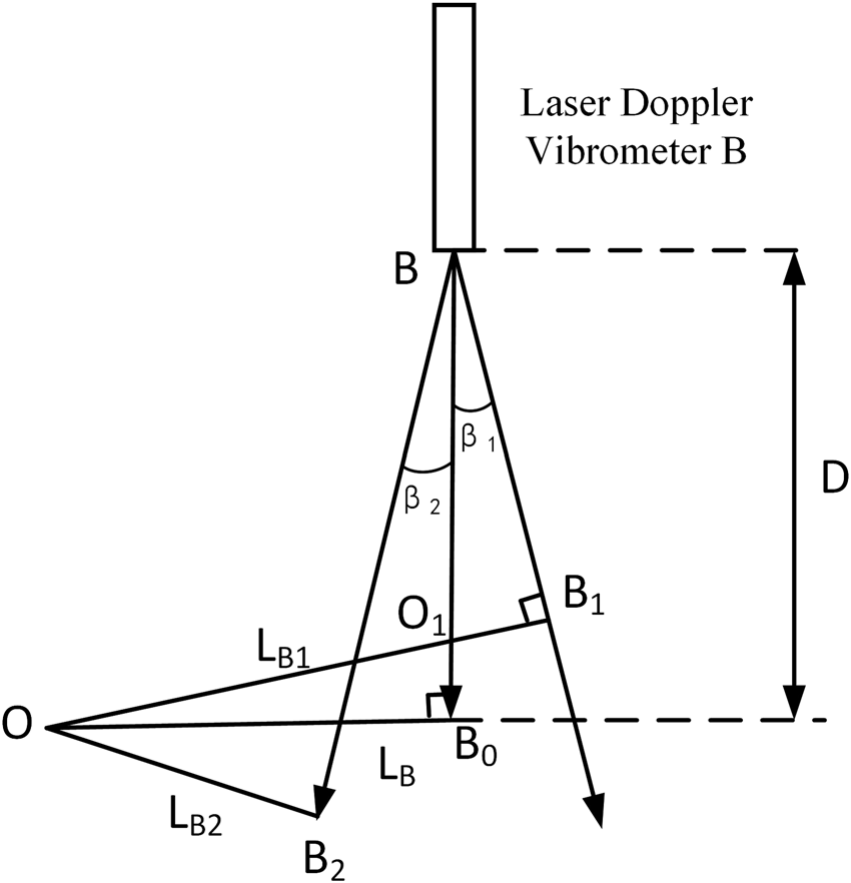
The angle measurement error mechanism caused by non-parallel angle of laser beams.

The system requires dual laser beams to be parallel to each other. From eq. 7, we can see that the instantaneous measurement velocity measured by laser Doppler vibrometer is only related to angular velocity $\omega$ and the distance from rotating center of object to laser beam ${L}_{1}$. When one of dual laser beams is not parallel to another, the distance from rotating center of object ${L}_{1}$ will change, thus the instantaneous measurement velocity measured by this non-parallel laser Doppler vibrometer will change, which eventually causes angular measurement error. Figure 3 shows the The angle measurement error mechanism that I’ve illustrated above. Please noted that, when we talk about non-parallel, there are two cases: the laser beam biased toward the rotation center (non-parallel angle ${\beta }_{2}$) and the laser beam away from rotation center (non-parallel angle ${\beta }_{1}$). ${L}_{B}$, ${L}_{B1}$, and ${L}_{B2}$ are the distance from rotation center to three laser beams respectively. Assuming D is the distance from laser emission point to OB_0_.

According to the geometric relationship in the figure, we have

14 $$\{\begin{array}{l}{L}_{B1}={L}_{B}\cdot cos{\beta }_{1}-D\cdot sin{\beta }_{1}\\ {L}_{B2}={L}_{B}\cdot cos{\beta }_{2}+D\cdot sin{\beta }_{2}\end{array}$$

From eq. 14, we can see that two different non-parallel condition show similar relationship with ${L}_{B}$ and D. We can specify that: when the laser beam the laser beam biased toward the rotation center, the non-parallel angle is positive; when the laser beam away from the rotation center, the non-parallel angle is negative. Thus, we can simplify equation (14) as

15 $${L}_{B1}({L}_{B2})={L}_{B}\cdot cos\beta +D\cdot sin\beta $$

where $\beta$ is the non-parallel angle of laser Doppler vibromter A. Substituting equation (15) into equation (9), we have angle measurement error caused by non-parallel of laser beams

16 $${\theta }_{p}=\theta -{\int }_{0}^{{t}_{0}}\frac{\omega }{L}\cdot ({L}_{A}+{L}_{B}cos\beta +Dsin\beta )dt$$

**Figure 4:**
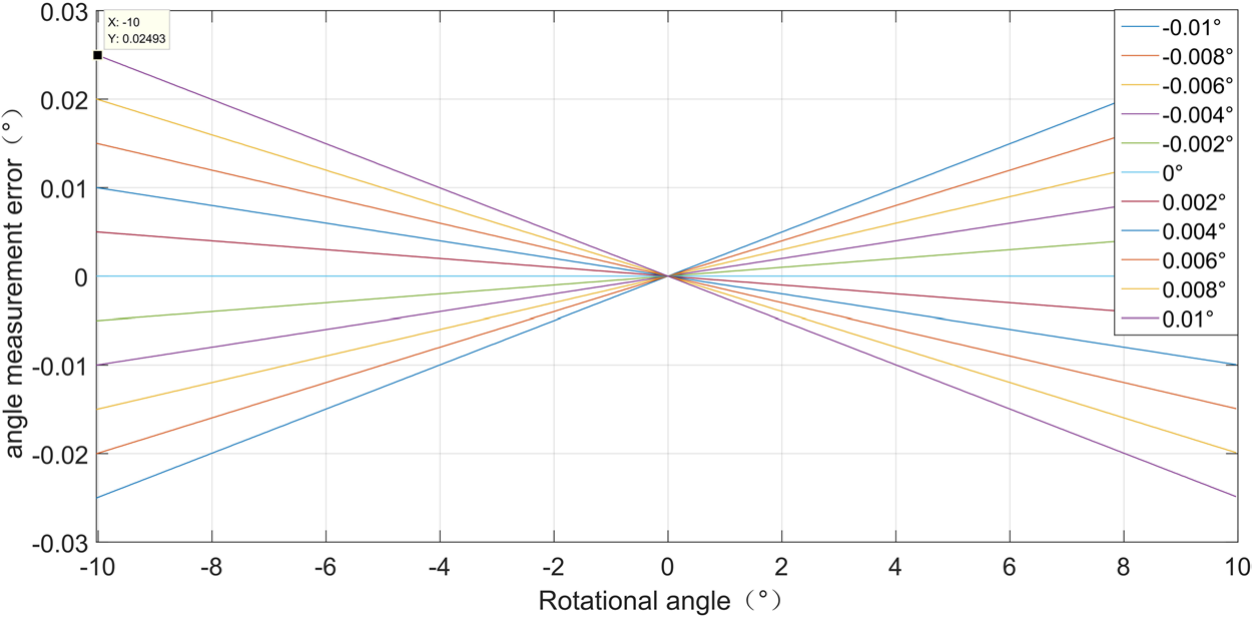
Simulation angular displacement error caused by non-parallel angle.

According to error equation (16), we simulate the relationship of the angular displacement error versus the rotational angle and the non-parallel angle as is shown in Fig. 4. One particular curve in this graph shows how the error increases with the rotational angle with a given non-parall angle. When the rotational angle increases, the measurement error increases synchronously in the presence of non-parallel angle. Different curve shows the influence of non-parallel angle to ADM error. Under a given rotational angle, the ADM error increases with the non-parallel angle. When the rotational angle is $-10^\circ$, and non-parallel angle is 0.01°, the ADM error is 0.025°. This result gives a clear illustration that if we try to get ADM accuracy higher than 0.01° under rotational angle of $\pm 10^\circ$, we have to reduce the non-parallel angle before measurement and try to compensate the error in data processing.

**Figure 5:**
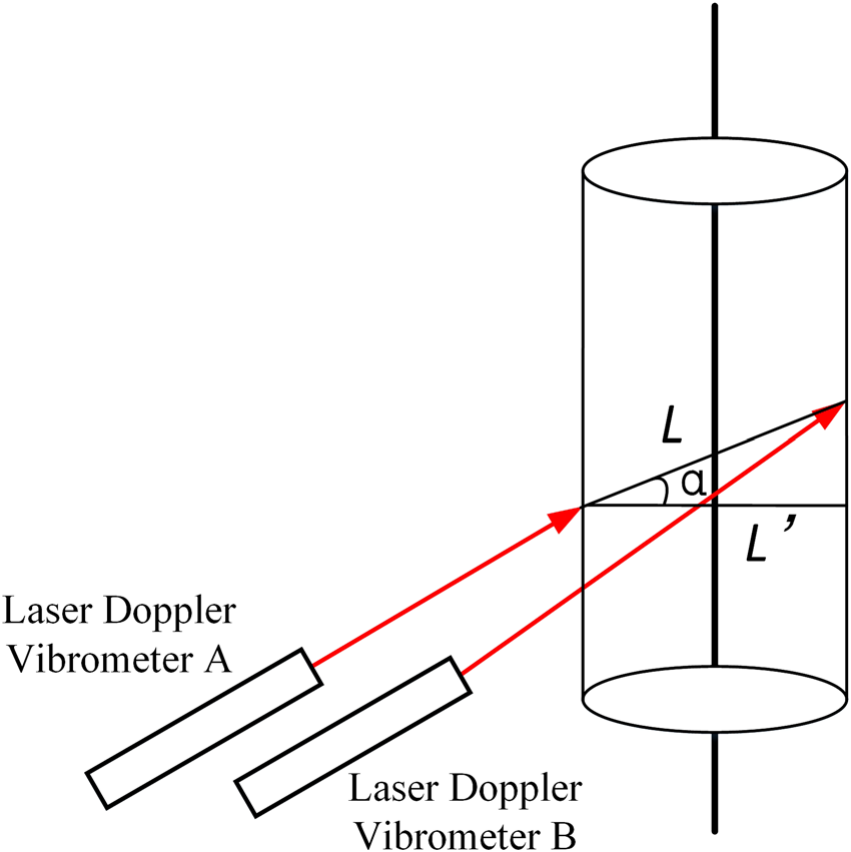
Non-coplanar measurement error schematic diagram.

Besides, the system requires the rotating axis of object be perpendicular to the plane where parallel beams are. When non-coplanar condition shown in Fig. 5 happens, the system will produce measurement errors. Assuming the angle between the plane where parallel beams are and the plane perpendicular to rotation axis is ${\rm{\alpha }}$, the actual spacing of the twin beams will become

17 $$L^{\prime} =L\ast cos\alpha $$

Thus, non-coplanar measurement error can be given by

18 $${\theta }_{c}=\frac{{\int }_{0}^{{t}_{0}}{v}_{A}dt-{\int }_{0}^{{t}_{0}}{v}_{B}dt}{L\cdot cos\alpha }-\frac{{\int }_{0}^{{t}_{0}}{v}_{A}dt-{\int }_{0}^{{t}_{0}}{v}_{B}dt}{L}\,$$

**Figure 6:**
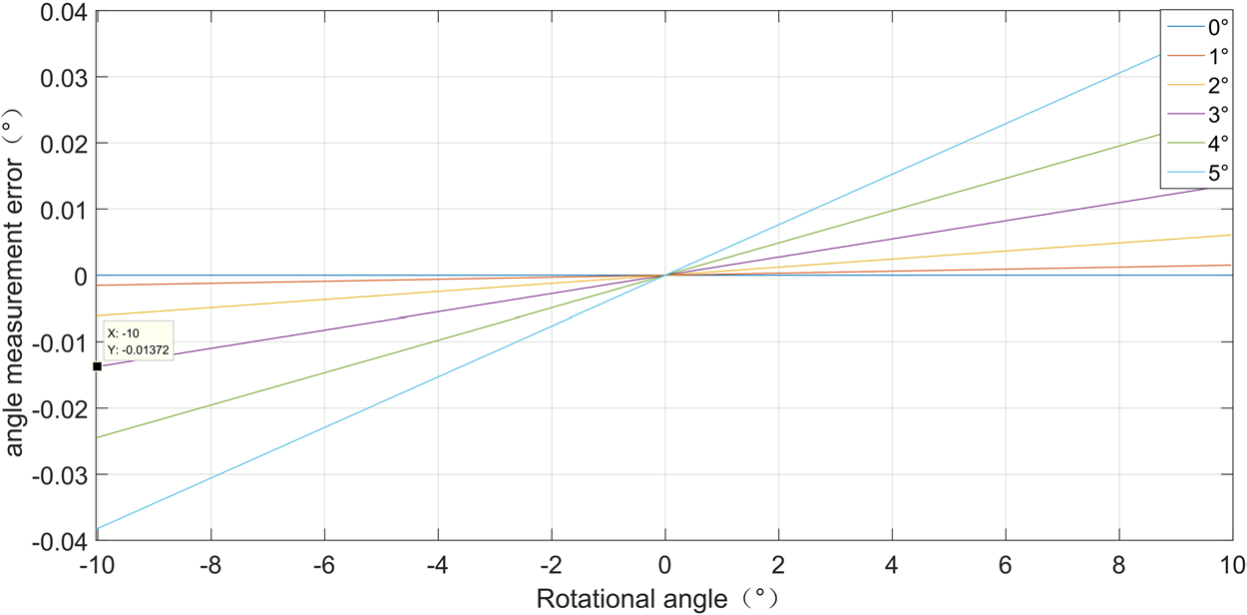
Simulation angular displacement error caused by non-coplanar angle.

According to equation (18), we simulate the relationship of the angular displacement error versus the rotational angle and the non-coplanar angle as is shown in Fig. 6. The result shows that the measurement error increases synchronously with rotation angle and non-coplanar angle. When the rotational angle is $-10^\circ$, and non-coplanar angle is 3°, the ADM error is 0.013°. This result gives a clear illustration that non-coplanar angle don’t have much very influence on ADM. Still, WE have to reduce reduce the non-coplanar angle under $-3^\circ$.

### Synchronization error

According to equation (9), the instantaneous rotational angular displacement is a function of velocity measured by d-LDVs and integration time. To get accurate instantaneous rotational angular displacement, the d-LDVs system have to ensure the synchronization of dual laser Doppler vibrometers. Time-delay between velocity measured by dual laser Doppler vibrometers will result in the ADM error. Suppose ${\rm{\Delta }}t$ is the time delay between ${v}_{A}$ and ${v}_{B}$, then we can get the instantaneous velocity measured by d-LDVs shown as below:

19 $$\{\begin{array}{l}{v}_{A}={v}_{A}(t)\\ {v}_{B}={v}_{B}(t+{\rm{\Delta }}t)\end{array}$$

Substituting equation (19) into equation (9) we can get the measured error caused by time-delay between dual laser Doppler vibrometers ${\rm{\Delta }}{{\rm{\theta }}}_{delay}$:

20 $${\rm{\Delta }}{{\rm{\theta }}}_{delay}=\frac{{\int }_{0}^{{t}_{0}}{v}_{A}(t)dt-{\int }_{0}^{{t}_{0}}{v}_{B}(t+{\rm{\Delta }}t)dt}{{\rm{L}}}-\frac{{\int }_{0}^{{t}_{0}}{v}_{A}dt-{\int }_{0}^{{t}_{0}}{v}_{B}dt}{{\rm{L}}}$$

**Figure 7:**
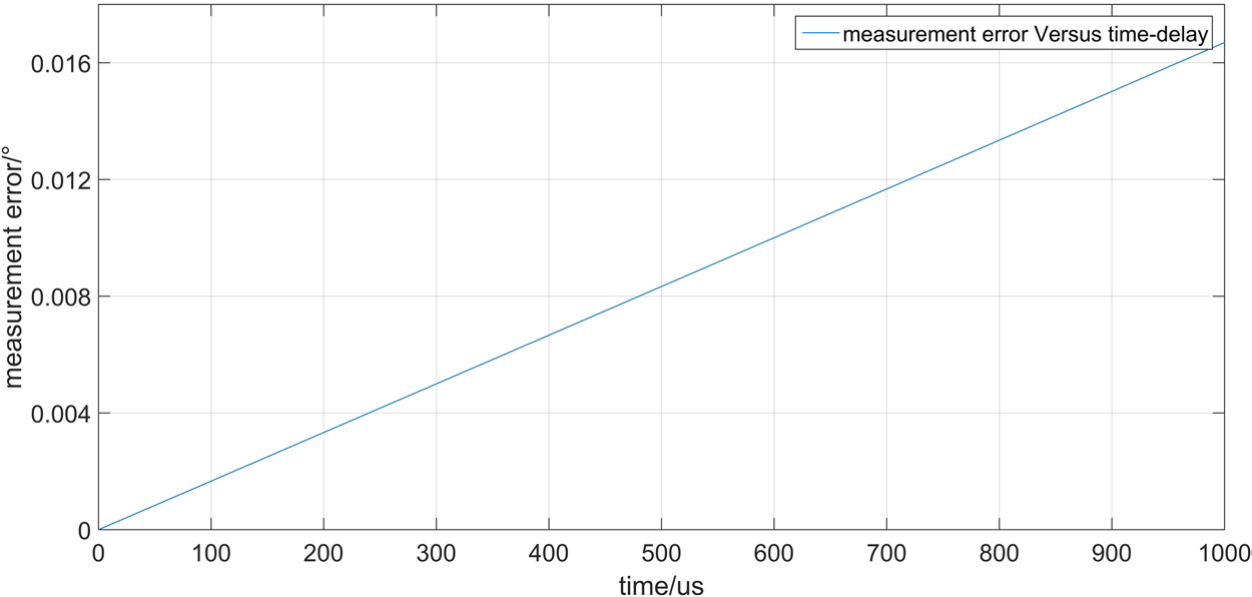
Simulation result of measurement error versus time –delay.

According to equation (20), we simulate the relationship between the measurement error and time-delay as is shown in Fig. 7.

In our simulation, sampling frequency is 2000 hz, maximum rotational angular displacement is 10°, sway frequency of target is 1 hz, time-delay between dual laser Doppler vibrometers change from 0 us-1 ms. As we can see in Fig. 7, ADM error increase with time-delay linearly. When the time-delay is 1000 us, the ADM error is 0.0167°. Simulation result shows that time-delay between dual laser Doppler vibrometers has great impact on the measurement result. In the actual measurement system, time-delay between dual laser Doppler vibrometers is less than 20 us, which corresponding to $(3.3\times {10}^{-4})^\circ$ angular measurement error according to simulation. Though time-delay has great impact on the measurement result, good synchronization between d-LDVs can overcome this measurement error.

### Sampling frequency caused error

The instantaneous rotational angular displacement of the object is calculated from the integration of velocity. Different numerical integration methods and integration period will result calculation error. As for numerical integration method, we use trapezoidal integration method. Integration period corresponds to the sampling frequency. Then, we will give the expression of measurement error caused by sampling frequency of system. The displacement of the moving target along the direction of laser can be given:

21 $$\{\begin{array}{c}{S}_{A}={\int }_{0}^{{t}_{0}}{v}_{A}dt\approx {\sum }_{i=l}^{n}(1/{f}_{sam})\frac{{v}_{A}({t}_{k})+{v}_{A}({t}_{k-1})}{2}\\ {S}_{B}={\int }_{0}^{{t}_{0}}{v}_{B}dt\approx {\sum }_{i=l}^{n}(1/{f}_{sam})\frac{{v}_{B}({t}_{k})+{v}_{B}({t}_{k-1})}{2}\end{array}$$

where ${f}_{sam}$ is the sampling frequency of system. Substituting equation (21) into equation (9), we can get the rotational angle calculated by numerical integration:

22 $${\theta }_{sam}=\frac{{\sum }_{i=1}^{n}\frac{{v}_{A}({t}_{k})+{v}_{A}({t}_{k-1})}{2\cdot {f}_{sam}}-{\sum }_{i=1}^{n}\frac{{v}_{B}({t}_{k})+{v}_{B}({t}_{k-1})}{2\cdot {f}_{sam}}}{{\rm{L}}}$$

Suppose the object sways in a sine wave ${\theta }_{real}$, then the measurement error caused by sampling frequency can be given:

23 $${{\rm{\Delta }}{\rm{\theta }}}_{sam}=\frac{{\sum }_{i=1}^{n}\frac{{v}_{A}({t}_{k})+{v}_{A}({t}_{k-1})}{2\cdot {f}_{sam}}-{\sum }_{i=1}^{n}\frac{{v}_{B}({t}_{k})+{v}_{B}({t}_{k-1})}{2\cdot {f}_{sam}}}{{\rm{L}}}-{\theta }_{real}$$

**Figure 8:**
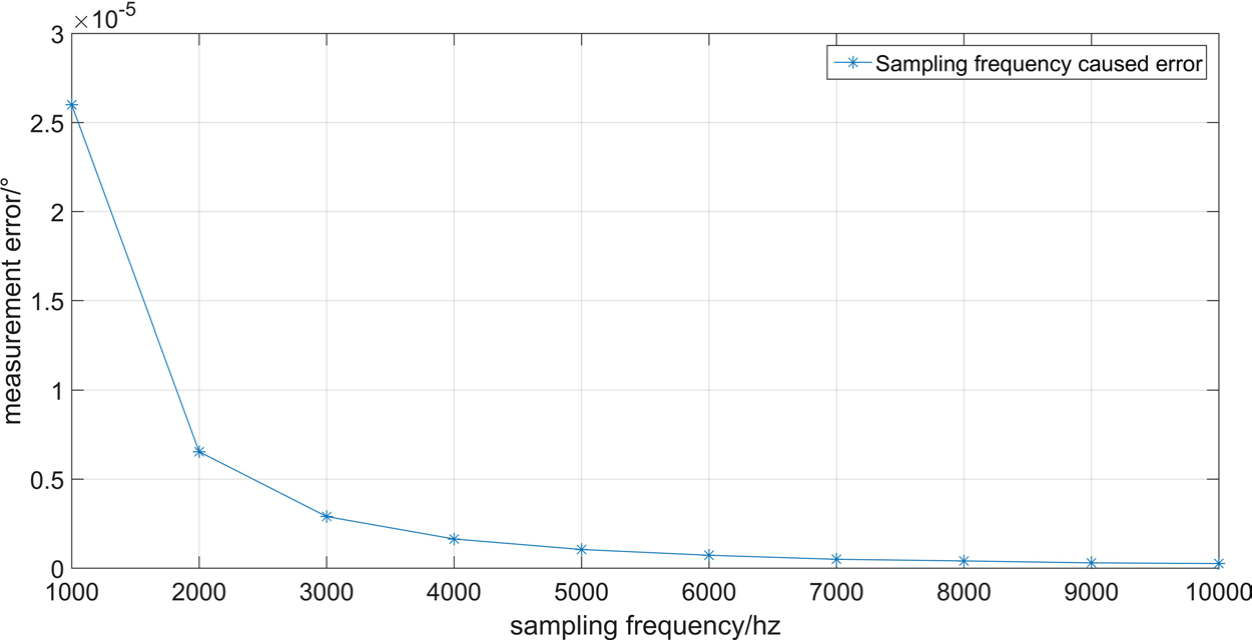
Sampling frequency caused error.

According to equation (23), we can simulate relationship between sampling frequency and measurement error. Figure 8 shows how measurement error change with sampling frequency. Measurement error decrease with sampling frequency. When the sampling frequency is 2000 hz, measurement error is $(6.54\times {10}^{-6})^\circ$. So, sampling frequency of system indeed have impact on the measurement error, but the measurement error is so small that we can ignore it.

## Calibration and Compensation

We have analyzed the measurement principle of the system and several major measurement errors. In the actual measurement process, these kinds of measurement errors coupled with the measurement uncertainty of the vibrometer itself will lead to larger errors in the angular displacement measurement results. So, we need to use high-precision angle standard to calibrate the measurement range, measurement accuracy and measurement error of the system. If the measurement error shows regularity, we can compensate it.

### Experimental setup

**Figure 9:**
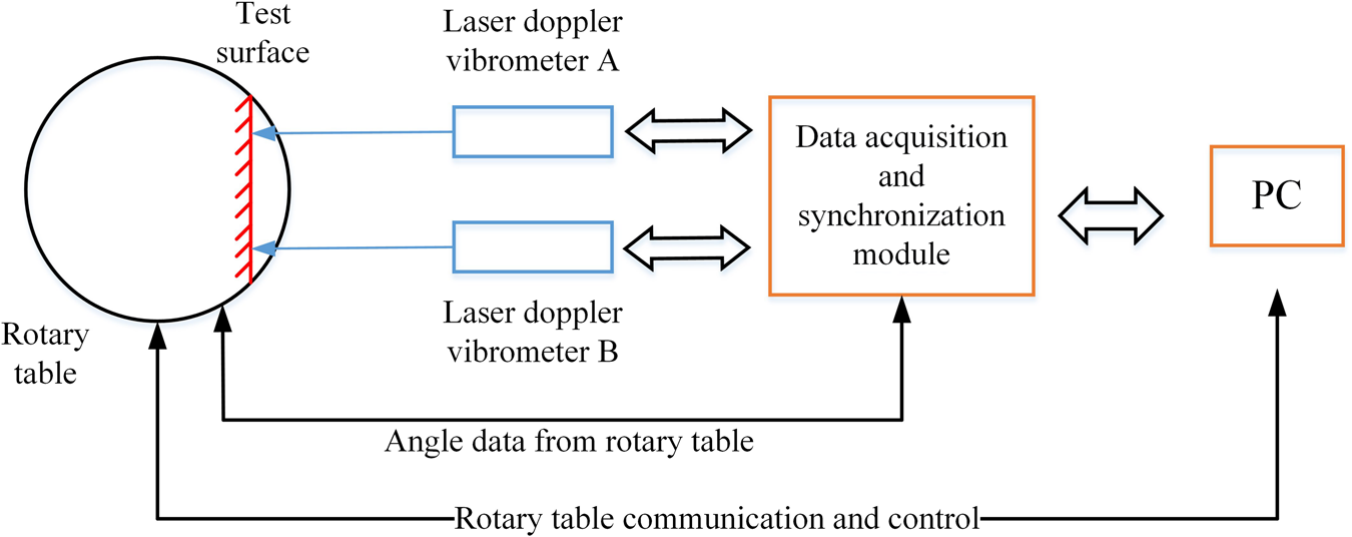
The hardware structure of the experimental setup.

The hardware structure of the experimental setup is shown in Fig. 9. The experiment system consists of two laser Doppler vibrometers, a rotary table, a test object, a data acquisition and synchronization module and a PC. Rotary table is used for providing rotational condition of test surface, as well as a high precision angle benchmark to calibrate angle measurement error of d-LDVs system. Angular displacement from the rotary table is calibrated by China Metrology Institute, the angular position measurement accuracy of the rotary table is 10″ (0.0028°), the angular position positioning repeatability of the rotary table is 3″ (0.00083°). It indicates that the angular displacement accuracy of rotary table is one order of magnitude higher than our proposed method and shows good stability. So we can use the angular displacement output by rotary table as an angular benchmark, and assume that the rotary table independent measurement is free from any error.

The test object is fixed on the rotational surface of the rotary table. Before measurement, a retro-reflective tape is stick on the surface of object, this retro-reflective tape is made by 3 M company to adapt environment of Low and uneven reflectivity of the target surface. The reflective of the retro-reflective tape is even and it can increase the diffuse reflection of red light. Under the control of PC, rotary table sways in a certain amplitude and frequency, the test object thus move with the rotary table at the same time. Laser Doppler vibrometers A and B measure the velocity of test surface during sway. Center wavelength of the laser Doppler vibrometer is 632.8 nm, velocity measurement range is 50 mm/s. D-LDVs are fixed on a tripod by which the lights emitted from the vibrometers are guaranteed parallel from each other. The distance between dual laser Doppler vibrometers is 70 mm. Sampling frequency of the data acquisition and synchronization module is 2000 Hz. Data acquisition and synchronization module collects the data of d-LDVs and the rotary table synchronously, and transfers the collected data to the PC. In the end, the PC will process the collected data and compare the calculated angular displacements from d-LDVs and rotary table.

### Primary experimental result

**Figure 10:**
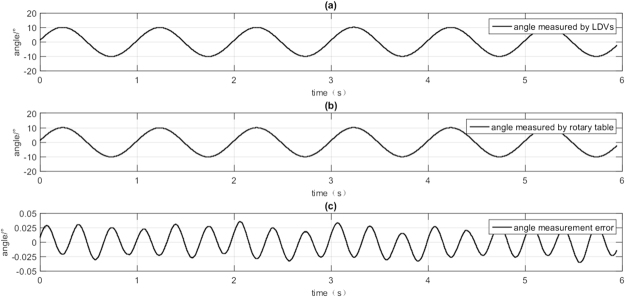
ADM result by the d-LDVs and the rotary table.

Rotary table sway at the frequency of 1 Hz and the angular displacement amplitude of 10°, Fig. 10(a,b) depicts the ADM result by d-LDVs and rotary table. It is shown that both of them have the same angular displacement response under the given movement. The ADM error of d-LDVs is obtained by doing subtraction between d-LDVs and the rotary table. Figure 10(c) presents the ADM error of d-LDVs, and the maximum error $|e|\,$is less than 0.0362°.

This is a rather good result of non-contact ADM. However, from Fig. 10, we can see that the ADM error and angular displacement measured by d-LDVs shows some kind of correlation. When the angular displacement measured by dual LDVs oscillates in a form of sine wave, the error oscillates in sine wave, but with different frequency.

**Figure 11:**
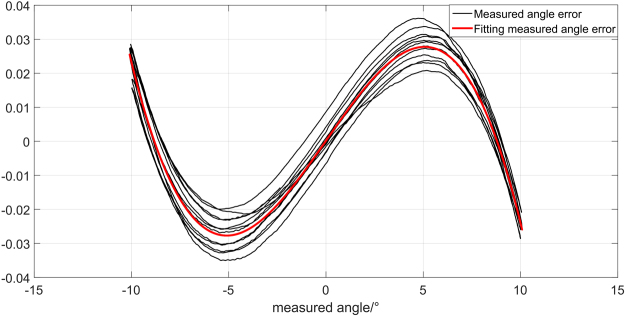
The relationship of ADM error and angular displacement measured by d-LDVs.

Taking the angular displacement measured by d-LDVs as X-axis and the error of d-LDVs as Y-axis, we can get the relationship between the ADM error and angular displacement measured by d-LDVs. The black line in Fig. 11 depicts the relationship of the ADM error with the angular displacement measured by d-LDVs in multiple measurement periods. The curves present in similar tracks, which means that the ADM error is caused by specific reasons and can be compensated.

### Compensation

From the analysis above, we can see that the system errors have a great influence on the measurement. In addition, environment conditions like temperature, humidity and magnetic field may impact the measurement accuracy and repeatability of d-LDVs. All these factors affect the measurement system simultaneously, resulting in the relationship between the ADM error and the angular displacement measured by d-LDVs depicted in Fig. 11. In order to get an accurate ADM result, it is necessary to compensate the system error. In this paper, we use polynomial fitting to compensate these errors, assuming the nonlinear error model is

24 $$f(x)=a{x}^{3}+b{x}^{2}+cx+d$$

where *x* is angular displacement measured by d-LDVs, *a, b, c* and *d* is the cubic coefficient, square coefficient, linear coefficient and constant coefficient respectively.

We take the data of black line shown in Fig. 11 into the error model above by the method of the least square. The fitting coefficients in equation (24) are then obtained as follows: *a* = −1.05e-4, *b* = −4.66e-6, *c* = 0.0082, *d* = 1.66e-4.

**Figure 12:**
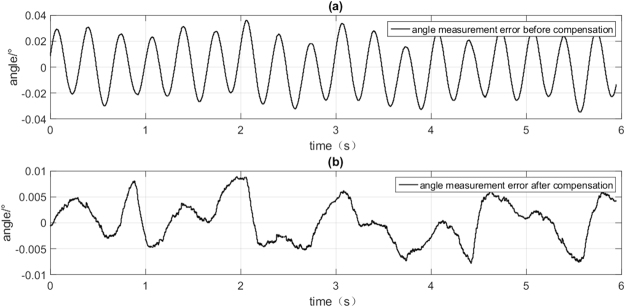
The comparison of ADM error before and after compensation.

After compensating the measurement error, Fig. 12 shows the comparison of the errors before and after compensation respectively. The result is obvious that the maximum ADM error $|e|$ can be reduced from 0.0362° to 0.0088°.

## Conclusion

Based on actual engineering needs, we propose this flexible, non-contact, high precision and large bandwidth angular displacement measurement system. Our proposed method for measuring rotational angular displacement adopts a pair of laser Doppler vibrometers. It is a flexible measurement system, researchers can adjust the parallel beam spacing and measurement distance according to the different measurement needs. Due to diffuse reflection, the measurement system do not need to contact the object, and a truly non-contact ADM has been realized. This method is insensitive to shape of object surface. The system can obtain angular displacement and translational displacement simultaneously from a composite motion of rotation and translation. Based on this algorithm and the actual situation, we have analyzed light intensity non-uniformity error, systematic defect error, synchronization error and sampling frequency caused error. In the meantime, we calibrate the measurement error of the system with a high-precision rotary table, fit the measurement error corresponding to each actual measurement angle and get fitted polynomial parameters. For each angle obtained, first use the parameters to calculate the fitted error, then use the angle value to subtract the fitting error, and we can get real angular displacement finally.

The measurement method proposed in this article has broad application prospects. Multiple laser Doppler vibrometers can be applied to real-time three-dimensional rotational angular displacement non-contact measurement of a moving object. It still need further study to realize real-time three dimensional rotational angular displacement non-contact measurement.

## References

[1] Jing F, Lin Y, Zhou Y, Zhang G (1992). Angular measurement by means of rotation of linear gratings. CIRP Annals-Manufacturing Technology.

[2] Zhang G, Wang C, Li Z (1994). Improving the accuracy of angle measurement system with optical grating. CIRP Annals-Manufacturing Technology.

[3] Palmer E (1988). Goniometer with continuously rotating gratings for use as an angle standard. Precision engineering.

[4] Nakano Y, Murata K (1986). Talbot interferometry for measuring the small tilt angle variation of an object surface. Applied optics.

[5] Wang A, Gill P, Molnar A (2009). Light field image sensors based on the Talbot effect. Applied optics.

[6] Wang, A., Gill, P. R. & Molnar, A. In *Solid-State Circuits Conference Digest of Technical Papers (ISSCC), 2011 IEEE International*. 412–414 (IEEE).

[7] Wang A, Molnar A (2012). A light-field image sensor in 180 nm CMOS. IEEE Journal of Solid-State Circuits.

[8] Dai X, Sasaki O, Greivenkamp JE, Suzuki T (1997). High accuracy, wide range, rotation angle measurement by the use of two parallel interference patterns. Applied optics.

[9] Filatov YV, Loukianov D, Probst R (1997). Dynamic angle measurement by means of a ring laser. Metrologia.

[10] Ikram M, Hussain G (1999). Michelson interferometer for precision angle measurement. Applied optics.

[11] Zhang G, Wang C, Hu X, Jing F, Hocken R (1987). A laser interferometric system for measuring arbritrary angles. CIRP Annals-Manufacturing Technology.

[12] Jin T, Xia G, Hou W, Le Y, Han S (2017). High resolution and stability roll angle measurement method for precision linear displacement stages. Review of Scientific Instruments.

[13] Ting, C., Liqiong, Z., Guizhen, Z. & Benyong, C. Design of a displacement/angle measurement system based on laser triangulation principle. (2006).

[14] Halliwell N, Pickering C, Eastwood P (1984). The laser torsional vibrometer: a new instrument. Journal of Sound and Vibration.

[15] Rothberg, S. *et al*. An international review of laser Doppler vibrometry: Making light work of vibration measurement. *Optics and Lasers in Engineering* (2016).

[16] Yeh Y, Cummins H (1964). Localized fluid flow measurements with an He–Ne laser spectrometer. Applied Physics Letters.

[17] Davis Q, Kulczyk W (1969). Vibrations of turbine blades measured by means of a laser. Nature.

[18] Di Maio D, Ewins D (2010). Applications of continuous tracking SLDV measurement methods to axially symmetric rotating structures using different excitation methods. Mechanical Systems and Signal Processing.

[19] Halliwell N (1996). The laser torsional vibrometer: a step forward in rotating machinery diagnostics. Journal of Sound and Vibration.

[20] Drew S, Stone B (1997). Torsional (rotational) vibration: Excitation of small rotating machines. Journal of sound and vibration.

[21] Collette C, Preumont A (2009). Laser measurement of torsional vibrations/longitudinal creepage of a railway wheel set on a scaled test bench. Optics and Lasers in Engineering.

[22] Drain, L. E. The laser Doppler techniques. *Chichester, Sussex, England and New York, Wiley-Interscience, 1980. 250 p*. (1980).

[23] Johansmann, M., Siegmund, G. & Pineda, M. Targeting the limits of laser Doppler vibrometry. *Proc. IDEMA*, 1–12 (2005).

[24] Diao X, Hu P, Xue Z, Kang Y (2016). High-speed high-resolution heterodyne interferometer using a laser with low beat frequency. Applied optics.

